# Dose-Response Effects of Acute Aerobic Exercise Duration on Cognitive Function in Patients With Breast Cancer: A Randomized Crossover Trial

**DOI:** 10.3389/fpsyg.2020.01500

**Published:** 2020-07-14

**Authors:** Elizabeth A. Salerno, Kendrith Rowland, Charles H. Hillman, Linda Trinh, Arthur F. Kramer, Edward McAuley

**Affiliations:** ^1^Cancer Prevention Fellowship Program, Division of Cancer Epidemiology & Genetics, Metabolic Epidemiology Branch, National Cancer Institute, Bethesda, MD, United States; ^2^Department of Kinesiology & Community Health, University of Illinois at Urbana-Champaign, Champaign, IL, United States; ^3^Carle Foundation Hospital, Urbana, IL, United States; ^4^College of Science, Northeastern University, Boston, MA, United States; ^5^Department of Kinesiology & Physical Education, University of Toronto, Toronto, ON, Canada; ^6^Beckman Institute for Advanced Science and Technology, University of Illinois at Urbana-Champaign, Champaign, IL, United States

**Keywords:** physical activity, breast cancer, cognition, dose-response, acute exercise

## Abstract

**Objective:**

To examine the differential effects of acute exercise duration on domains of executive function and processing speed in patients with breast cancer.

**Methods:**

Participants (*N* = 48, *M*_age_ = 56.02 ± 10.99) completed two sessions in counterbalanced order: moderate-intensity treadmill walking and sitting. Participants were also randomized to one of three duration conditions: 10 (*n* = 15), 20 (*n* = 16), or 30 (*n* = 17) min, reflecting the length of time spent walking and sitting. Immediately before and after each session, women completed a battery of cognitive tasks (e.g., inhibition, cognitive flexibility, spatial working memory, and processing speed).

**Results:**

Within- and between-subjects repeated-measures analyses of variance revealed time by condition interactions on both processing speed (*p* = 0.02) and spatial working memory (*p*s < 0.07), such that women demonstrated improved cognitive functioning regardless of the time spent walking. There were also several moderately sized three-way (e.g., time by condition by duration) interactions driven by declines in cognitive functioning after sitting on cognitive flexibility in the 10 (*d* = -0.96) and 30-min (*d* = -0.52) groups and inhibition in the 20-min group (*d* = 0.75). On the processing speed task, women performed significantly faster after walking compared with after sitting in the 20-min group (*d* = -0.24).

**Conclusions:**

For select cognitive domains, walking anywhere from 10 to 30 min is associated with significant benefits. Our findings suggest the need for further research into the mechanisms and dose–response relationships between acute exercise and cognition as well as how such acute bouts may be accumulated for larger, lasting cognitive benefits after breast cancer.

**Clinical Trial Registration:**

www.ClinicalTrials.gov, identifier NCT04255225

## Introduction

Approximately 75% of cancer patients experience some degree of cognitive deficit throughout their cancer experience with upward of one third of patients with breast cancer reporting impairments up to a decade after treatment (Koppelmans et al., 2012; Janelsins et al., 2014, 2017; Pendergrass et al., 2018). These impairments are most commonly reported within the domains of executive function, a family of executive control processes responsible for the selection, scheduling, and coordination of goal-directed behavior (Miyake et al., 2000; Diamond, 2013). Also known as cancer-related cognitive impairment (CRCI), cognitive dysfunction after cancer is attracting considerable attention among clinicians and scientists. The International Cognition and Cancer Task Force (ICCTF) (Wefel et al., 2011) has identified understanding, preventing, and improving CRCI as a priority for quality of life and longevity during cancer survivorship.

Physical activity has been consistently associated with improved cognition across the lifespan (Hillman et al., 2008). This behavior is protective against a host of diseases and has been shown to confer numerous health benefits to patients with breast cancer, such as reductions in cancer-related morbidity, recurrence, and all-cause mortality (Campbell et al., 2019; Patel et al., 2019). Recent work has tentatively highlighted chronic exercise training as a potential method of improving varying cognitive domains after breast cancer (Zimmer et al., 2016); however, cancer patients face a unique set of barriers (e.g., fatigue, pain, and symptomology) to initiating and maintaining long-term exercise regimens. The 2nd edition of the Physical Activity Guidelines for Americans has highlighted the robust benefits of acute exercise, or single sessions, for improving cognition across the lifespan (Piercy et al., 2018).

We recently reported on the beneficial effects of acute exercise on processing speed and spatial working memory in patients with breast cancer (Salerno et al., 2019), suggesting that acute bouts of physical activity may mitigate select domains of CRCI. Specifically, patients in this study demonstrated faster processing speed and trended toward faster and more accurate spatial working memory after 30 min of moderate-intensity walking compared to seated rest. This finding parallels those in previous studies in both healthy middle-aged and older adults (Chang et al., 2012) and individuals with varying memory complaints/impairments (Segal et al., 2012; Tsai et al., 2018). But half an hour of walking may be challenging to certain subgroups of patients, particularly those who are deconditioned or with significant barriers to longer walks such as peripheral neuropathy or osteoporosis. With a renewed focus on unbouted physical activity (Piercy et al., 2018), that cancer patients should “avoid inactivity” (American College of Sports Medicine, 2018), it is important to better understand the dose or volume of exercise responsible for providing patients with breast cancer with the greatest cognitive benefits (Mackenzie et al., 2016).

Limited as we are in our knowledge of the acute exercise–cognition relationship after cancer, evidence in middle-aged and older adults, both with and without health conditions, has highlighted the cognitive benefits of acute exercise (Chang et al., 2012). This literature further suggests that acute exercise bouts of a *moderate* length (e.g., 20 min) may result in greater improvements in cognitive functioning (Brisswalter et al., 2002; McMorris et al., 2011; Chang et al., 2012). Indeed, longer bouts of exercise may result in dehydration and depletion of energy storages that may contribute to cognitive decline (Brisswalter et al., 2002). Chang and colleagues (Chang et al., 2015) tested the dose-response relationship between exercise duration and cognition functioning whereby participants engaged in 10-, 20-, or 45-min bouts of exercise on a stationary cycle ergometer compared with seated reading. Twenty minutes of exercise produced the largest cognitive benefits, such that the relationship between exercise bout length and cognition was curvilinear.

The purpose of the current study was to examine the effects of varying durations of exercise (e.g., 10, 20, and 30 min) on cognitive function in patients with breast cancer to identify the optimal length of acute exercise. While metabolic and demographic differences may exist between previous samples and patients with breast cancer, previous moderator analyses of exercise duration focused primarily on younger samples (Brisswalter et al., 2002; Lambourne and Tomporowski, 2010; Chang et al., 2012). Thus, based on the existing literature, it was hypothesized that the exercise duration–cognition relationship in patients would mirror that of cancer-free adults (Chang et al., 2012). Specifically, participants who engaged in 20 min of exercise would demonstrate higher accuracy rates and faster reaction time on all cognitive domains from pre- to post-exercise compared with those who exercised for 10 and 30 min.

## Materials and Methods

### Participants, Procedures, and Design

**FIGURE 1:**
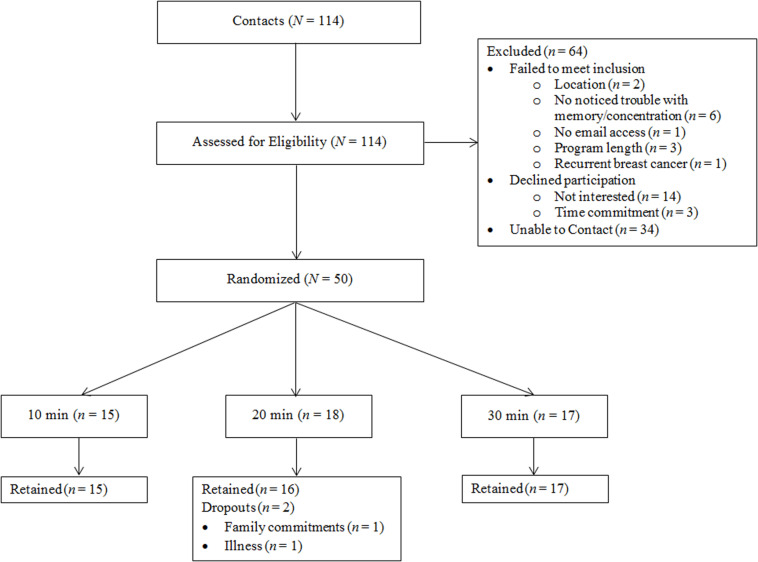
Participant flow through the study.

Patients with breast cancer (*N* = 48, *M*_age_ = 56.02 ± 10.99) were recruited locally from central Illinois to participate in a randomized, crossover study assessing the optimal length of acute exercise sessions for improved cognitive health. Recruitment, randomization, and all testing procedures were conducted on a rolling basis between September 2016 and February 2017, date of censoring. Recruitment methods utilized local media and family, friends, and oncologist referrals. Women were eligible to participate if they (i) were over the age of 18, (ii) had a past diagnosis of breast cancer (stages DCIS-III), (iii) completed primary treatment, (iv) were cleared for participation by their primary care physician or oncologist, (v) self-reported trouble with memory and/or concentration due to their breast cancer experience, and (vi) and had no other health reasons contraindicating exercise engagement. Figure 1 details participant flow through the study. All methods and procedures were approved by the institutional review board (IRB; ethics committee; approval #16912) at the University of Illinois at Urbana-Champaign, and written informed consent was obtained from all individual participants included in the study. This study is registered at Clinicaltrials.gov (NCT04255225).

Briefly, women in the current study completed one baseline appointment to orient participants to the cognitive tasks and two counterbalanced appointments: a walking session and a sitting session, both with pre- and post-cognitive testing. The order of the sessions and length of time spent walking and sitting (i.e., 10, 20, or 30 min) were determined randomly using block randomization (1:1 allocation ratio; block size of 9) after participants passed prescreening. Therefore, each patient completed both a walking and a sitting session in random order (within-subjects component) and were randomized to their session length (between-subjects component). The random allocation sequence was generated by the primary study investigator, and the first author enrolled and assigned participants to interventions.

### Acute Sessions

#### Walking (Exercise) Session

The walking session consisted of two rounds of cognitive tasks and either 10, 20, or 30 min spent walking on a treadmill, depending on group allocation. Upon arrival, participants completed a battery of cognitive tasks before being fitted with a heart rate monitor. They were then instructed to walk on the treadmill at 60% of their age-predicted maximal heart rate. Speed was selected by both the participant and supervising exercise specialist to ensure both comfort and appropriate intensity. Participants also maintained a rating of perceived exertion between 8 and 12 to account for varying heart rates that may have prevented target heart rate achievement (e.g., use of beta blocker medications) (Borg, 1998). Upon completion of the cool-down, participants completed the same battery of cognitive tasks.

#### Sitting (Control) Session

The sitting session was sequentially the same as the walking session with pre-/post-cognitive testing, but instead of walking, participants sat quietly in a distraction-free room for their respective duration (e.g., 10, 20, or 30 min). They were instructed to sit quietly, refrain from reading, talking, writing, and sleeping. All participants were given the option to watch a bland television show. Mean data (e.g., heart rate, blood pressure, and rating of perceived exertion) from the walking and sitting sessions are detailed in Table 1. Communication was standardized across sessions with talking kept to a minimum.

**TABLE 1:** Mean data from walking and sitting sessions.

	Walking Session	Sitting Session
	**Mean (SD) *n* = 46**	**Mean (SD) *n* = 48**
Heart rate (bpm)	100.0 (10.5)	76.5 (11.7)
Systolic blood pressure (mmHg)	133.2 (14.0)	116.6 (14.7)
Diastolic blood pressure (mmHg)	77.0 (7.0)	71.9 (13.2)
Rating of perceived exertion	9.2 (1.8)	7.3 (8.2)

*SD, standard deviation; bpm, beats per minute; mmHg, millimeter of mercury.*

### Cognitive Measures

The same cognitive battery was delivered immediately before and after each walking and sitting session. All instructions for the cognitive tasks were presented via computer screen or paper (depending on the task) for participants to read followed by a practice round with accuracy feedback. A trained staff member was present in the room during practices to answer questions and troubleshoot comprehension and/or computer difficulties. The staff member then exited the room before the actual trial began. Practice rounds were included pre-session and removed for all post-session assessments to ensure that potential acute effects were captured during the actual trial in the event of effect decay. The order of the tasks remained consistent: (1) flanker, (2) spatial working memory, (3) task switching, and (4) letter comparison. For each task, participants were encouraged to work as quickly but as accurately as possible.

#### Flanker

Designed to measure attention/inhibition, the flanker task was computer-based and required participants to focus on a cross in the middle of a black screen (Eriksen and Eriksen, 1974). Five arrows flashed on the screen before disappearing, and participants were asked to indicate the direction of the middle arrow using the keyboard (“X” for *left* or “M” for *right*). There were two separate conditions within this task: congruent and incongruent. A congruent condition had all five arrows pointed in the same direction, compared with an incongruent condition when the arrows pointed in different directions. Outcome variables were accuracy and reaction time.

#### Spatial Working Memory

A computer-based task, the spatial working memory paradigm, required participants to focus on a cross in the middle of a gray screen (Erickson et al., 2011); then 1, 2, or 3 black dots appeared for 500 ms before disappearing. A red dot then appeared for 2000 ms; participants were asked to indicate if the red dot location matched or did not match any of the previous black dot locations using the keyboard (“M” for *match* or “X” for *no match*). There were three separate trials corresponding to the number of black dots (1, 2, or 3). Outcome variables were accuracy and reaction time.

#### Task Switching

The task-switching paradigm was used to assess cognitive flexibility. This was a computer-based assessment that required participants to switch between sets based on the stimuli presented (Schneider and Logan, 2005). If a blue box was presented, participants had to indicate if the number inside the box was higher or lower than 5. If a pink box was presented, participants had to indicate if the number was odd or even. Participants completed a single task block for each condition. The third block was a mixed task block that presented a mix of blue and pink boxes in random order. Participants responded using the keyboard (“z” for *odd/low* or “/” for *high/even*). Variables from this paradigm included reaction time and accuracy. Within the mixed task block, outcome variables were also split by type of trial: switch and stay. When the presented trial was different from the preceding one (e.g., blue box followed by a pink box), it was a switch trial, compared with when the presented trial was the same as the preceding one (e.g., two pink boxes in a row), it was a stay trial.

#### Letter Comparison

Used to assess processing speed, the letter comparison measure was a paper-pencil based cognitive task that required participants to determine whether two strings of letters were the same or different (Salthouse, 1996). Participants were presented with two pages of several strings of consonants, each separated by a line (e.g., YSTX ____ YSTX). Participants marked an “S” on the line if the strings of letters were the same, or a “D” if they were different. Outcome variables from this task were averaged accuracy and reaction time across the two pages.

## Data Analysis

Thirty participants provided 80% power at alpha = 0.025 (Bonferroni-adjusted) to detect a moderately sized difference (η^2^ = 0.11), the smallest effect detected in our previous study (Salerno et al., 2019), between walking and sitting over time between the three different duration groups. It remains to be seen if such an effect size is clinically meaningful for cognition in this cancer cohort; however, this study is among the first to explore differential duration effects of acute exercise on cognition after cancer.

All analyses were conducted in SPSS (Version 24; IBM Corp. Armonk, NY) using a per-protocol approach. Descriptive statistics were used to assess normality as well as calculate means and standard deviations. We then conducted two (time; pre, post) by two (condition; walking, sitting) by three (duration; 10, 20, 30 min) repeated-measures analyses of variance to determine if changes in cognitive function over time between the walking and sitting conditions differed as a function of the condition duration. Cohen’s D effect sizes were also calculated. The threshold for significance was set to alpha < 0.05. Defined as the standardized difference between treatment and comparison group means (Cohen, 1988), effect sizes are independent of sample size and, thus, may be better indicators of group differences than traditional *p* values (Sullivan and Feinn, 2012). Given that the purpose of this project was to gauge the size of the effects of walking and sitting on cognitive function over time, we decomposed three-way interactions with a range of moderate effect sizes (i.e., η^2^ ≥ 0.07) to explore time by condition interactions within each of the three duration groups. It was hypothesized that moderately sized three-way effects would be driven by significant time and condition interactions favoring walking within the 20-min group. Forty-eight participants completed all three appointments. Cognitive accuracy scores lower than 50% (worse than chance) were considered participant error and winsorized. Missing data for health histories and demographic information was minimal (<10%) and, thus, treated as missing at random.

## Results

Participant demographic and cancer-specific characteristics are displayed in Table 2. Generally, participants were married (70.8%), Caucasian (85.4%), college educated (70.8%), and earning at least $75,000 per year (52.1%). Additionally, most participants underwent radiation (64.6%) or chemotherapy (66.7%) and had at least stage II malignancy (56.2%). No adverse events were reported.

**TABLE 2:** Participant demographic and breast-cancer specific characteristics.

	Full sample *N* = 48	10 min *n* = 15	20 min *n* = 16	30 min *n* = 17	*p* value
	
	Mean (SD) or %	Mean (SD) or %	Mean (SD) or %	Mean (SD) or %	
Age	56.02 (10.99)	56.80 (10.67)	55.13 (13.26)	56.18 (9.44)	0.92
Married	70.8%	60.0%	81.3%	70.6%	0.06
Employed full-time	43.8%	40.0%	56.3%	35.3%	0.41
Caucasian	85.4%	60.0%	93.8%	100%	0.003
≥College Graduate	70.8%	66.7%	68.8%	76.5%	0.63
Earning ≥$75,000/year	52.1%	40.0%	50.0%	64.7%	0.31
Disease stage					0.38
<Stage 2	39.6%	46.7%	37.5%	35.3%	
≥Stage 2	56.2%	53.3%	62.5%	52.9%	
Unknown	4.2%	0%	0%	11.8%	
Received chemotherapy	66.7%	53.3%	68.8%	76.5%	0.57
Months since chemotherapy	49.6 (38.4)	38.4 (22.1)	64.7 (52.8)	43.7 (29.4)	0.27
Received radiation	66.7%	60.0%	62.5%	76.5%	0.91
Months since radiation	48.7 (40.1)	36.9 (22.1)	67.1 (59.2)	42.7 (27.9)	0.21
Body mass index	29.0 (6.4)	29.0 (7.2)	32.0 (6.9)	27.0 (4.2)	0.09
Depressive symptoms	5.5 (4.4)	6.0 (5.0)	4.0 (4.0)	6.4 (4.0)	0.51
Anxiety symptoms	5.5 (3.6)	5.0 (3.0)	5.9 (4.0)	6.0 (3.0)	0.35
Quality of life	145.1 (16.1)	145.0 (15.0)	145.0 (18.0)	145.0 (16.0)	0.99
Fatigue	52.3 (10.0)	51.0 (12.0)	53.0 (9.0)	53.0 (9.0)	0.85
Predicted VO2max	23.3 (4.7)	22.0 (4.2)	22.0 (5.4)	25.0 (4.1)	0.08

*SD, standard deviation.*

### Flanker

Three-way RM-ANOVAs revealed a moderately sized three-way interaction between time, condition, and duration on incongruent flanker accuracy [*F*(2,42) = 2.57, *p* = 0.09, η^2^ = 0.11]. Exploratory decomposition of this interaction within duration groups revealed no significant interactions between time and condition (*p*s > 0.20). However, there was a large effect size in the 20-min duration group (*d* = 0.75) with slower performance after sitting compared with maintenance post-walking. The three-way interaction for incongruent reaction time was non-significant with a negligible effect size (*p* = 0.51, η^2^ = 0.03). No meaningful three-way interactions emerged for accuracy or reaction time on the congruent task. Analyses also revealed a significant main effect of time for reaction time (*p* = 0.001) on the incongruent task such that women performed significantly slower over time regardless of whether they walked or sat.

### Spatial Working Memory

No significant three-way interactions emerged; therefore, duration groups were collapsed across the sample. Two-way RM-ANOVAs in the full sample revealed a significant time by condition interaction for reaction time on 2-dot trials [*F*(1,45) = 4.81, *p* = 0.03, *η*^2^ = 0.10]. This interaction was driven by significantly shorter reaction time (i.e., faster performance) after exercise compared with rest irrespective of assigned duration group (*d* = -0.12). Analyses also revealed a time by condition interaction that trended toward significance for accuracy on 3-dot trials [*F*(1,44) = 3.54, *p* = 0.07, *η^2^* = 0.07], explained by improved accuracy after exercise compared with rest (*d* = 0.18).

### Task Switching

Analyses also revealed a moderately sized three-way interaction for reaction time on the single task block [*F*(2,44) = 11.87, *p* = 0.21, η^2^ = 0.07]. Further exploratory decomposition revealed a significant time by condition interaction in the 10-min duration group [*F*(1,13) = 13.56, *p* = 0.003, η^2^ = 0.51] and an interaction approaching significance in the 30-min duration group [*F*(1,16) = 3.19, *p* = 0.09, η^2^ = 0.17]. These findings were explained by slower performance after sitting in these groups (*d* = -0.96) and (*d* = -0.52), respectively. The time by condition interaction in the 20-min duration group mirrored this trend, but the interaction was non-significant (*p* = 0.44, *d* = -0.23). The three-way interaction for accuracy on the single task block was non-significant with a negligible effect size (*p* = 0.87, η^2^ = 0.01). No significant three-way interactions emerged for accuracy or reaction time on either the mixed stay or switch blocks. Analyses further revealed a significant main effect of time for both accuracy and reaction time (*p*s < 0.01) on the single task block such that women were more accurate but slower over time across walking and sitting conditions. There was also a significant time by condition interaction for reaction time (*p* = 0.001), driven by significantly slower performance after the sitting condition regardless of duration group.

### Letter Comparison

Three-way RM-ANOVAs revealed a moderately sized three-way interaction for reaction time on the processing speed task [*F*(2,45) = 1.60, *p* = 0.21, η^2^ = 0.07]. Upon decomposing this interaction within duration groups, there was a significant time by condition interaction in the 20-min duration group [*F*(1,15) = 6.43, *p* = 0.04, η^2^ = 0.26] driven by significantly faster performance after walking compared with after sitting (*d* = -0.24). No significant or meaningful time by condition interactions emerged in either the 10- or 30-min duration groups (*p*s > 0.20, *d*s < -0.10). The three-way interaction for accuracy was non-significant with a negligible effect size (*p* = 0.41, η^2^ = 0.04). Analyses also revealed a significant main effect of time for accuracy (*p* < 0.04) driven by increased accuracy over time regardless of activity condition or duration group. In addition, there was a significant time by condition interaction across the full sample for reaction time [*F*(1,47) = 5.84, *p* = 0.02, η^2^ = 0.11], such that women performed significantly faster after walking regardless of how long they walked. Means (SE) and effect sizes for significant outcomes are displayed in Table 3.

**TABLE 3:** Means (SE) for cognitive tasks.

Measure	Pre	Post	*d*
**Task switching single task**			
Accuracy (%)			
Overall			
Walking	0.95 (0.02)	0.98 (0.01)	0.09
Sitting	0.96 (0.01)	0.98 (0.00)	
Reaction time (ms)			
10 min			
Walking	754.49 (33.37)	791.60 (41.13)	–0.96
Sitting	775.64 (39.35)	944.20 (47.19)	
20 min			
Walking	702.10 (20.76)	736.10 (33.66)	–0.23
Sitting	782.38 (47.42)	850.70 (34.83)	
30 min			
Walking	700.71 (15.61)	746.90 (36.11)	–0.52
Sitting	785.14 (37.77)	893.13 (44.71)	
**Spatial Working Memory 2-dot**			
Accuracy (%)			
Overall			
Walking	0.90 (0.02)	0.90 (0.03)	–0.15
Sitting	0.91 (0.03)	0.90 (0.02)	
Reaction time (ms)			
Overall			
Walking	840.58 (20.48)	805.15 (21.48)	–0.12
Sitting	817.22 (19.90)	798.92 (18.68)	
10 min			
Walking	887.58 (35.96)	864.18 (32.62)	–0.06
Sitting	866.56 (35.59)	851.76 (27.70)	
20 min			
Walking	814.39 (28.64)	761.81 (32.84)	–0.35
Sitting	781.93 (26.89)	767.93 (27.74)	
30 min			
Walking	822.23 (32.50)	791.30 (29.72)	–0.05
Sitting	804.81 (28.96)	780.35 (26.23)	
**Spatial Working Memory 3-dot**			
Accuracy (%)			
Overall			
Walking	85.7(0.01)	87.0 (0.01)	0.18
Sitting	86.5(0.01)	86.2 (0.01)	
**Flanker Incongruent**			
Accuracy (%)			
10 min			
Walking	0.97 (0.01)	0.95 (0.02)	–0.33
Sitting	0.93 (0.03)	0.94 (0.03)	
20 min			
Walking	0.97 (0.01)	0.97 (0.01)	0.75
Sitting	0.98 (0.01)	0.96 (0.01)	
30 min			
Walking	0.97 (0.01)	0.96 (0.01)	–0.02
Sitting	0.96 (0.01)	0.95 (0.01)	
Reaction time (ms)			
Overall			
Walking	630.97 (11.33)	651.13 (14.12)	0.07
Sitting	641.10 (16.35)	658.59 (15.36)	
**Processing Speed**			
Accuracy (%)			
Overall			
Walking	0.94 (0.01)	0.95 (0.01)	0.07
Sitting	0.95 (0.01)	0.96 (0.01)	
Reaction time (ms)			
Overall			
Walking	63.34 (3.90)	60.57 (3.90)	–0.10
Sitting	63.05 (4.22)	61.88 (4.18)	
10 min			
Walking	69.30 (4.19)	67.87 (4.33)	–0.01
Sitting	70.26 (4.95)	69.03 (4.96)	
20 min			
Walking	61.31 (3.21)	57.96 (3.31)	–0.24
Sitting	60.14 (3.23)	59.94 (3.50)	
30 min			
Walking	59.41 (4.31)	55.88 (4.07)	–0.08
Sitting	58.74 (4.49)	56.67 (4.09)	

*SE, standard error; ms, milliseconds.*

## Discussion

The present study was designed to determine if the effects of walking versus sitting on cognitive function in patients with breast cancer differed as a function of walking duration (i.e., 10 vs. 20 vs. 30 min). To our knowledge, this is the first study to examine the dose-response relationship between acute exercise duration and cognition in patients with breast cancer. Analyses revealed that regardless of how long they walked, patients performed significantly faster on processing speed and spatial working memory tasks post-exercise compared with post-sitting. The capacity of a walk spanning anywhere from 10 to 30 min to confer cognitive benefits is encouraging when we consider the number of barriers cancer patients must surmount in their return to daily life post-treatment; a short walk is an achievable target for a wide range of patients relative to function and fitness. These results are consistent with those of our previous study in 27 patients with breast cancer (Salerno et al., 2019) in which we observed benefits in the domains of processing speed and spatial working memory after 30 min of walking. The current findings replicate and extend this work through evidencing cognitive benefits after shorter walks and highlighting potential avenues of exploration for the dose-response relationship between acute exercise and cognition post-cancer. Of course, both studies were conducted in small samples of homogeneous patients with breast cancer, and more research is needed before formally recommending acute exercise for cognition during cancer survivorship; however, physical activity benefits are far-reaching, and these results support its utility for improved health after cancer.

Of further interest were moderately sized three-way interactions for inhibition accuracy and cognitive flexibility reaction time, driven by significantly slower performance after *sitting* (inhibition accuracy: 20-min; cognitive flexibility: 10- and 30-min) rather than faster performance after walking. Historically, dose-response effects in non-diseased populations have been driven by faster performance after acute exercise (Tomporowski, 2003; McMorris et al., 2008; McMorris and Hale, 2012); however, more recent work in children and young adults has highlighted the detrimental effects of prolonged sitting time on cognition (Drollette et al., 2012; Pontifex et al., 2015). Our findings may be the result of screen time during the sitting session (Takeuchi et al., 2015), where almost 90% of the current sample opted to watch television. Given the rise in incentivized sedentary behaviors in the 21st century (e.g., desk jobs and delivered goods) and high prevalence of screen time in our daily lives, it is encouraging that a short bout of exercise has the capacity to at least maintain select domains of cognitive functioning.

The present findings then juxtapose two separate considerations: the beneficial effects of exercise and detrimental effects of sitting. It appears that for cognitive flexibility and inhibition, sitting has a more robust, negative effect than acute exercise does a positive effect, whereas the opposite can be said for processing speed and spatial working memory. This selectivity is similar to that seen in cancer-free adults (Colcombe and Kramer, 2003; Colcombe et al., 2006; Hillman et al., 2008; Erickson et al., 2011), and may be explained through distinct biological mechanisms. It is generally accepted that exercise results in increased concentrations of circulating brain-derived neurotrophic factor, endothelial growth factor, and insulin-like growth factor (Lista and Sorrentino, 2010). These factors are thought to work synergistically in increasing neurogenesis in distinct areas of the brain such as the hippocampus, which in turn has been related to improvements in spatial memory in older adults (Erickson et al., 2011). However, this mechanistic literature has yet to be clarified with conflicting findings across studies. A better understanding of the biological/psychosocial mechanisms underpinning the acute exercise-cognition relationship in cancer patients specifically is warranted to highlight targetable pathways and better inform the design of behavioral interventions. Just as physical activity is distinct from sedentary behavior, future work may seek to better understand these differential acute effects. This may include the use of active control groups or different sedentary activities during sitting sessions.

These mixed findings may be the product of the cognitive tests employed. Discordance in the literature between subjective (e.g., survey and interview) and objective (e.g., neuropsychological tests) measures of CRCI (Pullens et al., 2010; Janelsins et al., 2014) suggests that patients with breast cancer suffer impairments in cognition that we may not fully understand and subsequently fail to test. One solution would be adopting a transdisciplinary approach with input from behavioral, neurological, and physiological scientists to develop more appropriate tests of CRCI (Horowitz et al., 2018). We must also consider that an acute bout of walking is simply not powerful enough to meaningfully improve certain cognitive domains after breast cancer to the extent that chronic exercise engagement might. Given the differential cognitive and affective effects (Hopkins et al., 2012) and mechanisms (Pesce, 2012) between acute and chronic exercise, it will be important to characterize the domains most amenable to acute bouts of exercise compared to those requiring sustained, consistent engagement in physical activity over time. This would allow for the design and delivery of physical activity programs that maximize cognitive benefits while also minimizing unnecessary burden.

The present study should be interpreted within the context of its strengths and weaknesses. It is the first, to our knowledge, to examine the dose-response relationship between exercise duration and cognitive function in patients with breast cancer using an attainable, easily implemented behavior: walking. Additionally, the cognitive tasks employed were well-validated, reliable measures of cognitive function across the lifespan; however, more work should be conducted to validate such measures in cancer populations specifically as well as ascertain if such measures adequately measure CRCI. The main limitation of the present study is the small sample size (i.e., <20 subjects per group), which was comprised of primarily Caucasian, highly educated women, limiting generalizability of the results. However, if the effect sizes reported herein can be replicated in a larger sample, such findings would have important public health implications. We also note that several analyses were conducted in the full sample of 48 patients, highlighting no difference between the walking durations for spatial working memory and processing speed. Further, these findings are only relevant to four domains of cognitive function. Future research should use a variety of tasks in randomized order to ensure that the full effects of exercise on cognition are not lost to task specificity and/or the possible transient effects of acute exercise. Finally, blinding was not possible due to the design of this study and may have introduced bias given the known benefits of physical activity after cancer. However, we employed objective cognitive tasks, potentially reducing the risk of bias that may have presented itself with self-reported outcomes.

Findings from the present study suggest that exercise’s influence on cognition in patients with breast cancer is selective by length of time spent walking and sitting, response outcome (i.e., accuracy or reaction time) and cognitive task type. While the optimal length of exercise for eliciting the greatest cognitive benefits remains unclear, our results suggest that walking from 10 to 30 min may provide significant short-term benefits for select cognitive domains. Given the meaningful effect sizes herein, future research efforts should focus on elucidating these effects in larger trials of heterogeneous cancer patients as well as how such acute bouts may be accumulated for larger, lasting cognitive benefits after breast cancer.

## Data Availability Statement

The raw data supporting the conclusions of this article will be made available by the authors, without undue reservation.

## Ethics Statement

The studies involving human participants were reviewed and approved by University of Illinois Institutional Review Board (IRB). The patients/participants provided their written informed consent to participate in this study.

## Author Contributions

ES, CH, LT, AK, and EM contributed to conception and design of the study. KR recruited participants. ES collected the data, performed the statistical analysis, and wrote the first draft of the manuscript. All authors contributed to manuscript revision, read, and approved the submitted version.

## Conflict of Interest

The authors declare that the research was conducted in the absence of any commercial or financial relationships that could be construed as a potential conflict of interest.

## References

[B1] American College of Sports Medicine (2018). *Guidelines for Exercise Testing and Prescription - American College of Sports Medicine*, 10th Edn Alphen aan den Rijn: Wolters Kluwer.

[B2] Borg G. (1998). *Borg’s Perceived Exertion and Pain Scales.* Champaign, IL: Human Kinetics.

[B3] Brisswalter J. Collardeau M. René A. (2002). Effects of acute physical exercise characteristics on cognitive performance [Internet]. *Sports Med.* 32 555–566. 10.2165/00007256-200232090-00002 12096929

[B4] Campbell K. L. Winters-stone K. M. Wiskemann J. May A. M. Schwartz A. L. Courneya K. S. (2019). Exercise guidelines for cancer survivors: consensus statement from international multidisciplinary roundtable exercise guidelines for cancer survivors: consensus statement from International Multidisciplinary Roundtable SPECIAL COMMUNICATIONS. *Med. Sci. Sport Exerc.* 51 2375–2390. 10.1249/mss.0000000000002116 31626055 PMC8576825

[B5] Chang Y.-K. Chu C.-H. Wang C.-C. Wang Y.-C. Song T.-F. Tsai C.-L. (2015). Dose-response relation between exercise duration and cognition. *Med. Sci. Sports Exerc.* 47 159–165. 10.1249/mss.0000000000000383 24870572

[B6] Chang Y. K. Labban J. D. Gapin J. I. Etnier J. L. (2012). The effects of acute exercise on cognitive performance: a meta-analysis. *Brain Res.* 1453 87–101. 10.1016/j.brainres.2012.02.068 22480735

[B7] Cohen J. (1988). *Statistical Power Analysis for the Behavioral Sciences.* Hillsdale, NJ: Larence Earlbaum Associates, 20–26.

[B8] Colcombe S. Kramer A. F. (2003). Fitness effects on the cognitive function of older adults: a Meta-Analytic study. *Psychol. Sci.* 14 125–130. 10.1111/1467-9280.t01-1-01430 12661673

[B9] Colcombe S. J. Erickson K. I. Scalf P. E. Kim J. S. Prakash R. McAuley E. (2006). Aerobic exercise training increases brain volume in aging humans. *J. Gerontol. A Biol. Sci. Med. Sci.* 61 1166–1170. 10.1093/gerona/61.11.1166 17167157

[B10] Diamond A. (2013). Executive functions. *Annu. Rev. Psychol.* 64 135–168. 23020641 10.1146/annurev-psych-113011-143750 PMC4084861

[B11] Drollette E. S. Shishido T. Pontifex M. B. Hillman C. H. (2012). Maintenance of cognitive control during and after walking in preadolescent Children. *Med. Sci. Sport Exerc.* 44 2017–2024. 10.1249/mss.0b013e318258bcd5 22525770

[B12] Erickson K. I. Voss M. W. Prakash R. S. Basak C. Szabo A. Chaddock L. (2011). Exercise training increases size of hippocampus and improves memory. *Proc. Natl. Acad. Sci. U.S.A.* 108 3017–3022. 10.1073/pnas.1015950108 21282661 PMC3041121

[B13] Eriksen B. A. Eriksen C. W. (1974). Effects of noise letters upon the identification of a target letter in a nonsearch task. *Percept. Psychophys.* 16 143–149. 10.3758/BF03203267

[B14] Hillman C. H. Erickson K. I. Kramer A. F. (2008). Be smart, exercise your heart: exercise effects on brain and cognition. *Nat. Rev. Neurosci.* 9 58–65. 10.1038/nrn2298 18094706

[B15] Hopkins M. E. Davis F. C. VanTieghem M. R. Whalen P. J. Bucci D. J. (2012). Differential effects of acute and regular physical exercise on cognition and affect. *Neuroscience* 215 59–68. 10.1016/j.neuroscience.2012.04.056 22554780 PMC3374855

[B16] Horowitz T. S. Suls J. Treviño M. (2018). A call for a neuroscience approach to cancer-related cognitive impairment. *Trends Neurosci.* 41 493–496. 10.1016/j.tins.2018.05.001 29907436

[B17] Janelsins M. C. Heckler C. E. Peppone L. J. Kamen C. Mustian K. M. Mohile S. G. (2017). Cognitive complaints in survivors of breast cancer after chemotherapy compared with age-matched controls: an analysis from a nationwide, multicenter, prospective longitudinal study. *J. Clin. Oncol.* 35 506–514. 10.1200/JCO.2016.68.5826 28029304 PMC5455314

[B18] Janelsins M. C. Kesler S. R. Ahles T. A. Morrow G. R. (2014). Prevalence, mechanisms, and management of cancer-related cognitive impairment. *Int. Rev. Psychiatry* 26 102–113. 10.3109/09540261.2013.864260 24716504 PMC4084673

[B19] Koppelmans V. Breteler M. M. B. Boogerd W. Seynaeve C. Gundy C. Schagen S. B. (2012). Neuropsychological performance in survivors of breast cancer more than 20 years after adjuvant chemotherapy. *J. Clin. Oncol.* 30 1080–1086. 10.1200/jco.2011.37.0189 22370315

[B20] Lambourne K. Tomporowski P. (2010). The effect of exercise-induced arousal on cognitive task performance: a meta-regression analysis. *Brain Res.* 1341 12–24. 10.1016/j.brainres.2010.03.091 20381468

[B21] Lista I. Sorrentino G. (2010). Biological mechanisms of physical activity in preventing cognitive decline [Internet]. *Cell. Mol. Neurobiol.* 30 493–503. 10.1007/s10571-009-9488-x 20041290 PMC11498799

[B22] Mackenzie M. J. Zuniga K. E. McAuley E. (2016). “Cognitive impairment in breast cancer survivors: the protective role of physical activity, cardiorespiratory fitness, and exercise training,” in *Exercise-Cognition Interaction: Neuroscience Perspectives*, Ed. McMorris T. (Cambridge, MA: Academic Press), 399–419. 10.1016/b978-0-12-800778-5.00019-0

[B23] McMorris T. Collard K. Corbett J. Dicks M. Swain J. P. (2008). A test of the catecholamines hypothesis for an acute exercise–cognition interaction. *Pharmacol. Biochem. Behav.* 89 106–115. 10.1016/j.pbb.2007.11.007 18164752

[B24] McMorris T. Hale B. J. (2012). Differential effects of differing intensities of acute exercise on speed and accuracy of cognition: a meta-analytical investigation. *Brain Cogn.* 80 338–351. 10.1016/j.bandc.2012.09.001 23064033

[B25] McMorris T. Sproule J. Turner A. Hale B. J. (2011). Acute, intermediate intensity exercise, and speed and accuracy in working memory tasks: a meta-analytical comparison of effects. *Physiol. Behav.* 102 421–428. 10.1016/j.physbeh.2010.12.007 21163278

[B26] Miyake A. Friedman N. P. Emerson M. J. Witzki A. H. Howerter A. Wager T. D. (2000). The unity and diversity of executive functions and their contributions to complex “Frontal Lobe” tasks: a latent variable analysis. *Cogn. Psychol.* 41 49–100. 10.1006/cogp.1999.0734 10945922

[B27] Patel A. V. Friedenreich C. M. Moore S. C. Hayes S. C. Silver J. K. Campbell K. L. (2019). Introduction: the American College of sports medicine convened an inter-national multidisciplinary roundtable on exercise. *Med. Roundtable Rep. Phys. Act* 51 2391–2402. 10.1249/MSS.0000000000002117 PMC6814265 31626056

[B28] Pendergrass J. C. Targum S. D. Harrison J. E. (2018). Cognitive impairment associated with cancer: a brief review. *Innov. Clin. Neurosci.* 15 36–44. 29497579 PMC5819720

[B29] Pesce C. (2012). Shifting the focus from quantitative to qualitative exercise characteristics in exercise and cognition research. *J. Sport Exerc. Psychol.* 34 766–786. 10.1123/jsep.34.6.766 23204358

[B30] Piercy K. L. Troiano R. P. Ballard R. M. Carlson S. A. Fulton J. E. Galuska D. A. (2018). The physical activity guidelines for Americans. *JAMA* 320:2020. 10.1001/jama.2018.14854 30418471 PMC9582631

[B31] Pontifex M. B. Parks A. C. Henning D. A. Kamijo K. (2015). Single bouts of exercise selectively sustain attentional processes. *Psychophysiology* 52 618–625. 10.1111/psyp.12395 25523887 PMC4398582

[B32] Pullens M. J. J. De Vries J. Roukema J. A. (2010). Subjective cognitive dysfunction in breast cancer patients: a systematic review. *Psychooncology* 19 1127–1138. 10.1002/pon.1673 20020424

[B33] Salerno E. A. Rowland K. Kramer A. F. McAuley E. (2019). Acute aerobic exercise effects on cognitive function in breast cancer survivors: a randomized crossover trial. *BMC Cancer* 19:371. 10.1186/s12885-019-5589-1 31014267 PMC6480426

[B34] Salthouse T. A. (1996). The processing-speed theory of adult age differences in cognition. *Psychol. Rev.* 103 403–428. 10.1037/0033-295x.103.3.403 8759042

[B35] Schneider D. W. Logan G. D. (2005). Modeling task switching without switching tasks: a short-term priming account of explicitly cued performance. *J. Exp. Psychol. Gen.* 134 343–367. 10.1037/0096-3445.134.3.343 16131268

[B36] Segal S. K. Cotman C. W. Cahill L. F. (2012). Exercise-induced noradrenergic activation enhances memory consolidation in both normal aging and patients with amnestic mild cognitive impairment. *J. Alzheimer’s Dis.* 32 1011–1018. 10.3233/jad-2012-121078 22914593 PMC3951984

[B37] Sullivan G. M. Feinn R. (2012). Using effect size-or why the p value is not enough. *J. Grad. Med. Educ.* 4 279–282. 10.4300/jgme-d-12-00156.1 23997866 PMC3444174

[B38] Takeuchi H. Taki Y. Hashizume H. Asano K. Asano M. Sassa Y. (2015). The impact of television viewing on brain structures: cross-sectional and longitudinal analyses. *Cereb. Cortex* 25 1188–1197. 10.1093/cercor/bht315 24256892

[B39] Tomporowski P. D. (2003). Effects of acute bouts of exercise on cognition. *Acta Psychol.* 112 297–324. 10.1016/s0001-6918(02)00134-8 12595152

[B40] Tsai C. L. Ukropec J. Ukropcová B. Pai M. C. (2018). An acute bout of aerobic or strength exercise specifically modifies circulating exerkine levels and neurocognitive functions in elderly individuals with mild cognitive impairment. *Neuroimage Clin.* 17 272–284. 10.1016/j.nicl.2017.10.028 29527475 PMC5842646

[B41] Wefel J. S. Vardy J. Ahles T. Schagen S. B. (2011). International Cognition and Cancer Task Force recommendations to harmonise studies of cognitive function in patients with cancer. *Lancet Oncol.* 12 703–708. 10.1016/s1470-2045(10)70294-1 21354373

[B42] Zimmer P. Baumann F. T. Oberste M. Wright P. Garthe A. Schenk A. (2016). Effects of exercise interventions and physical activity behavior on cancer related cognitive impairments: a systematic review. *Biomed. Res. Int.* 2016:1820954. 10.1155/2016/1820954 PMC4842032 27144158

